# Role of automated insulin delivery (AID) systems in glucose control in patients with diabetes mellitus undergoing dialysis in Calabria: AID-DIAL-CAL

**DOI:** 10.1007/s00592-026-02690-9

**Published:** 2026-04-15

**Authors:** Elena Succurro, Giuseppe Cersosimo, Paola Sarnelli, Francesco Brisinda, Ilaria Gattuso, Valeria Mazza, Giuseppe Fabiano, Fiorella Iorio, Roberta Arena, Giovanni Mazzitello, Ramona Nicotera, Maria Capria, Gianluigi Zaza, Michele Andreucci, Raffaele Mancini, Francesco Andreozzi

**Affiliations:** 1 Department of Medical and Surgical Sciences, University Magna Graecia of Catanzaro, Viale Europa, Catanzaro, 88100 Italy; 2 Research Center for the Prevention and Treatment of Metabolic Diseases (CR METDIS), University Magna Graecia of Catanzaro, Catanzaro, Italy; 3 Azienda Ospedaliera Annunziata Cosenza, Cosenza, Italy; 4 ASL Viterbo, Viterbo, Italy; 5 ASP Catanzaro, Catanzaro, Italy; 6 Department of Civil Engineering, University of Calabria, Rende, Italy

**Keywords:** Automated insulin delivery system, End-stage kidney disease, Dialysis, Time in range, Diabetes mellitus, Quality of Life

## Abstract

**Aims:**

To describe the main glycemic outcomes and the Quality of Life (QOL) observed in a cohort of people with type 1 (T1D) or type 2 (T2D) insulin-treated diabetes under dialysis who started an Automated Insulin Delivery (AID) system.

**Methods:**

This is a longitudinal retrospective pilot real-world analysis of 14 individuals with T1D and T2D undergoing dialysis who began using an AID system to optimize glycemic control. All subjects used the MiniMed™ 780G system. Glucose metrics were collected at baseline, 3, 6, and 12 months after initiating the SmartGuard™ feature. The WHOQoL-Bref questionnaire was administered at the last follow-up to evaluate the QOL.

**Results:**

Out of the 14 people, 8 reached 1-year follow-up. Time in Range (TIR) increased from 63% at baseline to 69% at 12 months, Time Below Range < 70 mg/dL (TBR70) decreased from 0.3% to 0%, and Time Above Range > 250 mg/dL (TAR250) decreased from 6.7% to 3.9%. Seven out of eight subjects who reached a 12-month follow-up achieved all three glycemic targets for this fragile population (TIR > 50%, TBR70 < 1% and TAR250 < 10%). At the last follow-up, 58.3% of the users were satisfied or very satisfied with their health status, versus only 25% with the previous treatment, and 81.7% had a good or very good QOL, whereas only 8.3% had a good QOL, and no one had a very good QOL with the previous treatment.

**Conclusion:**

This pilot real-world study showed how the use of an AID system is safe and can help to improve the glycemic outcomes and the QOL of people with diabetes in dialysis.

## Introduction

Diabetes mellitus is one of the leading causes of chronic kidney disease (CKD), with approximately 30 to 40% of people living with diabetes developing CKD [1–3]. In the adult population, diabetes mellitus remains a primary cause of end-stage kidney disease (ESKD), a condition characterized by the irreversible failure of kidney function, necessitating dialysis or transplantation for survival [1, 3].

Glycaemic management in patients with ESKD is challenging because of defects in glucose and insulin metabolism due to uremia; decreased gluconeogenesis and impaired insulin clearance by the kidney, resulting also in greater susceptibility to iatrogenic hypoglycaemia; impaired counterregulatory hormone responses (i.e. cortisol, growth hormone); increased erythrocyte glucose uptake during hemodialysis; and the underlying mild inflammatory state, which may predispose patients to hyperglycaemia [4, 5].

People with diabetes mellitus and ESKD have a higher risk of developing severe hypoglycaemic events and hyperglycaemic crises requiring emergency room visits and/or hospitalisation than individuals at high risk non-ESKD [4]. Hypoglycaemic episodes are associated with a higher risk of recurrent hypoglycaemia and mortality after initiation of dialysis and also impose a significant burden of care [6].

Automated insulin delivery (AID) systems have revolutionized diabetes care, particularly in people with type 1 diabetes (T1D) [3]. Meta-analyses undertaken from trial data show that AID systems outperform non-automated systems with improvements in time spent in the target glucose range of approximately 8–12% points, reduced time spent in hyperglycaemia, reduced mean glucose and either a reduction or no increase in time in hypoglycaemia [3, 7]. Extensive evidence supports the benefits of current AID systems in improving glycaemic management, decreasing hypoglycaemia risk and fear of hypoglycaemia, and improving Quality of Life (QOL) for people with diabetes mellitus [3, 7–14].

A recent Clinical Practice Consensus document recommends the use of AID in patients with chronic renal failure, even if the evidence on the use of these devices in people with diabetes mellitus and ESKD is still limited [15]. Two small studies conducted in adults with type 2 diabetes (T2D) receiving haemodialysis showed significant improvements in Time in range (TIR) without increasing the risk of hypoglycaemia in patients in treatment with AID compared with conventional insulin therapy [16, 17]. These small studies demonstrate the glycaemic benefit of AID in adults with T2D undergoing haemodialysis, but further investigations are needed in this population, as well as in T1D population receiving haemodialysis and in individuals receiving peritoneal dialysis.

The primary objectives of this longitudinal retrospective pilot real-world study were to describe the glycaemic outcomes and the QOL observed in a cohort of patients with diabetes mellitus undergoing dialysis using AID. The secondary outcome was to describe the AID settings used in subjects undergoing dialysis.

## Materials and methods

### Study design and setting

This is a retrospective multicentric real-world analysis of anonymized data of T1D or T2D insulin-treated individuals undergoing dialysis, who began AID therapy to optimize glycaemic control. The pilot real-world study was conducted at the Department of Medical and Surgical Sciences of the University “Magna Graecia” of Catanzaro, the ASP of Catanzaro (Italy), and the Annunziata hospital in Cosenza (Italy). AID therapy was proposed to T1D or T2D insulin -treated subjects undergoing hemodialysis or peritoneal dialysis, without a history of dementia or cognitive impairment. Other therapy exclusion criteria were non-self-sufficient or elderly people (> 75 years) without a dedicated caregiver, subjects with only basal insulin (< 10 units/day), recent hospitalization for acute events, presence of acute infections or malignant diseases. All subjects in the analysis were treated with the MiniMed™ 780G system for at least 3 months.

All the individuals received education on diabetes management and on the use of the AID system, accurately assessed and periodically reassessed by diabetologists.

This retrospective analysis was approved by the Regional Territorial Ethics Committee (Comitato Etico Territoriale Regione Calabria, approval code: 213/2025) and was conducted in accordance with the 1964 Declaration of Helsinki and its subsequent amendments.

### Onboarding and follow-up protocol

Subjects with sub-controlled diabetes undergoing hemodialysis or peritoneal dialysis were identified by nephrologists and referred to diabetologists for consultation. After being deemed suitable for AID therapy, they were first educated on using CGM only (Medtronic Guardian™ 4 sensor) at the dialysis clinics. After two weeks, they were re-evaluated and, if deemed ready, started using the insulin pump. For 14 days, they used the manual mode, followed by activation of the SmartGuard™ feature. In all users, the following system settings were used: glucose target = 100 mg/dL and AIT = 2.30 h, Patients were then followed up at the dialysis clinics at 3, 6, and 12 months by diabetologists.

The same protocol had been followed for subjects who underwent peritoneal dialysis, but the diabetological ambulatories were used instead of the dialysis ones.

### Laboratory determinations

HbA1c was measured with high-performance liquid chromatography using an NGSP-certified automated analyzer (Adams HA-8160 HbA1c analyzer, Menarini, Italy).

### Glucose metrics

Glucose data were extracted from Medtronic CareLink™ Clinic, a dedicated web-cloud platform (https://carelink.minimed.eu/app/login).

Glucose metrics, such as mean sensor glucose (SG), TIR (70–180 mg/dL), TBR (< 70 mg/dL), TAR (> 180 mg/dL), TAR180 (180–250 mg/dL), TAR250 (> 250 mg/dL), and GMI, were collected at baseline, 3, 6, and 12 months after initiating the SmartGuard™ feature. The baseline was considered a 14-day run-in period during which the subjects used only the CGM.

### Quality of Life

At the last follow-up, the validated Italian version of the WHOQoL-Bref questionnaire was administered to assess the perceived QOL in this fragile population. This questionnaire was chosen because it compares the current therapies with the previous one and measures changes across time in the impact of disease and impairment on daily activities.

### Statistical analysis

Descriptive statistics were used to summarize results. These include mean and SD for continuous variables and counts and percentages for categorical variables. Summary statistics were reported with a maximum of 2 decimal places, as appropriate. Mean changes for each pairwise period comparison were estimated using linear mixed models, to account for the within-patient correlation. Estimates along with their 95% Confidence Intervals (CIs) are provided. SAS software, version 9.4, (SAS Institute Inc., Cary, NC, USA) was used to perform all statistical analyses.

## Results

Twenty adult subjects were started on CGM and among these, fourteen started AID therapy and were included in this analysis. Six refused an insulin pump or were not suitable for AID and continued to use CGM only.

Out of the 14 people, 8 reached the 12-month follow-up. The remaining 6 subjects were enrolled at a later date, and the timing of their enrolment in the study did not allow them to reach the 12-month follow-up in the data analyses.

The mean age was 60.6 years, with females comprising 21.4% (*n* = 3) of the cohort. The average duration of diabetes was 21.9 years. Most of the subjects were affected by T2D (85.7%). The average HbA1c was 7.6% and the average BMI was 29.7 kg/m^2^. Most of them had at least one complication: in particular, 71.4% of the subjects had a history of cardiovascular disease; 50% had retinopathy: and 21.4% had diabetic foot. An estimated 28.6% of the subjects in the study had peripheral artery disease and 21.4% had a history of major amputation. Of these, 71.4% had hypertension and 57.1% had dyslipidemia. Most of the subjects had a secondary school diploma (63.7%) and most of them had a caregiver (71.4%). Thirteen subjects underwent hemodialysis, and one underwent peritoneal dialysis. The average duration of dialysis was 4.3 years, and the majority followed a three-times-week dialysis rhythm (92.3%). All baseline characteristics are summarized in Table 1. During the follow-up, no hospitalization, acute diabetes-related complications or adverse events were reported among the patients in study.

**Table 1:** Baseline characteristics of the subjects in study

	Subjects (*N* = 14)
Age, years	60.6 ± 10.7
	41.0–75.0
Female, *n* (%)	3 (21.4%)
Duration of diabetes (yrs.)	21.9 ± 10.9 6.0–42.0
*Type of diabetes*	
Type 1, *n* (%)	2 (14.3%)
Type 2, *n* (%)	12 (85.7%)
Basal HbA1c (%)	7.6 ± 0.8
BMI (Kg/m^2^)	29.7 ± 7.7
Cardiovascular disease, *n* (%)	10 (71.4%)
Dyslipidemia, *n* (%)	8 (57.1%)
Hypertension, *n* (%)	10 (71.4%)
Diabetic foot, *n* (%)	3 (21.4%)
Peripheral Artery Disease, *n* (%)	4 (28.6%)
Major amputation, *n* (%)	3 (21.4%)
Retinopathy, *n* (%)	7 (50.0%)
Caregiver, *n* (%)	10 (71.4%)
*Education*	
Primary school, *n* (%)	4 (28.6%)
Secondary school, *n* (%)	9 (63.7%)
Tertiary school, *n* (%)	1 (7.7%)
Duration of dialysis, years	4.3 ± 2.6
	1.0–12.0
*Type of dialysis*	
Hemodialysis, *n* (%)	13 (92.9%)
Peritoneal, *n* (%)	1 (7.1%)
*Hemodialysis rhythm*	
Twice a week, *n* (%)	1 (7.7%)
Three times a week, *n* (%)	12 (92.3%)

### Glycemic outcomes

**Fig. 1:**
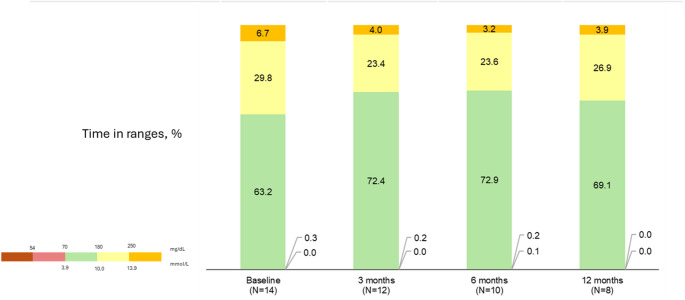
Glycemic outcomes in subjects undergoing dialysis using the AID system over time

Fourteen subjects with diabetes mellitus undergoing dialysis using the AID system exhibited improvement in glycemic outcomes over time. Interestingly, we observed clinically meaningful improvements in glycemic parameters after just 3 months of use of the AID system. Notably, patients exhibited a clinically relevant increase of TIR from 63% at baseline to 72.4% at 3 months (mean change 9.4% [95% CI − 1.1, 19.8]), and a reduction of TAR from 36.5% to 27.4% (mean change − 9.3% [95% CI − 19.7, 1.2]), TAR180 from 29.8% to 23.4% (mean change − 6.5% [95% CI −14.2, 1.2]), TAR 250 from 6.7% to 4% (mean change − 2.7% [95% CI − 7.6, 2.2]), and TBR from 0.3% to 0.2% (mean change − 0.1% [95% CI − 0.4, 0.1]) (Fig. 1; Tables 2 and 3).

**Table 2:** Glycemic outcomes in the subjects in study with AID system over time

	Baseline	3-months	6-months	12-months
	*N* = 14	*N* = 14	*N* = 10	*N* = 8
Sensor use (%)	81.3 ± 20.3	93.7 ± 4.9	88.8 ± 12.6	95.5 ± 2.2
Mean sensor glucose (mg/dl)	169.9 ± 19.1	156.9 ± 21.3	158 ± 10.7	161 ± 20.3
CV (%)	26.6 ± 5	26.5 ± 3.9	28.8 ± 6.5	25.5 ± 3.9
GMI (%)	7.4 ± 0.5	7.1 ± 0.5	7.1 ± 0.3	7.2 ± 0.5

**Table 3:** Mean glycemic outcomes changes in the subjects in study after AID system use over time

	Mean change 3-months post SmartGuard^TM^activation vs. baseline period (95% CI)	Mean change 6-months post SmartGuard^TM^activation vs. baseline period (95% CI)	Mean change 12-months post SmartGuard^TM^activation vs. baseline period (95% CI)
Sensor use (%)	11.8 (2.7; 20.9)	6.7 (− 0.9; 14.4)	13.0 (2.0; 24.0)
Mean SG (mg/dl)	− 13.2 (− 26.8; 0.5)	− 13.8 (− 26.2; − 1.4)	− 10.0 (− 26.0; 6.0)
CV (%)	− 0.2 (− 4.1; 3.8)	1.1 (− 2.7; 4.9)	− 1.3 (− 5.7; 3.2)
GMI (%)	− 0.3 (− 0.6; 0.0)	− 0.3 (− 0.6; − 0.0)	− 0.2 (− 0.6; 0.1)
Time (%) in < 54 mg/dL	− 0.0 (−0.1; 0.0)	0.0 (− 0.0; 0.1)	− 0.0 (− 0.1; 0.0)
Time (%) in 54–69 mg/dL	− 0.1 (− 0.3; 0.1)	− 0.1 (− 0.3; 0.1)	− 0.2 (− 0.5; − 0.0)
Time (%) in < 70 mg/dL	− 0.1 (− 0.4; 0.1)	− 0.1 (− 0.3; 0.1)	− 0.3 (− 0.5; 0.0)
Time (%) in 70–180 mg/dL	9.4 (− 1.1; 19.8)	10.9 (1.4; 20.4)	6.7 (− 5.5; 18.9)
Time (%) in 181–250 mg/dL	− 6.5 (− 14.2; 1.2)	− 6.5 (−13.8; 0.8)	− 3.1 (− 11.9; 5.8)
Time (%) in > 250 mg/dL	− 2.7 (− 7.6; 2.2)	− 4.1 (− 8.7; 0.4)	− 3.2 (− 8.9; 2.5)
Time (%) in > 180 mg/dL	− 9.3 (− 19.7; 1.2)	− 10.8 (− 20.4; − 1.2)	− 6.4 (− 18.7; 5.8)

Mean changes for each pairwise period comparison were estimated using linear mixed models, to account for the within-patient correlation. The estimates mean changes are presented as mean along with their 95% Confidence Intervals (CIs)

Furthermore, after 3 months of AID treatment, patients showed a decrease of GMI from 7.4% at baseline to 7.1% (mean change − 0.3% [95% CI − 0.6, 0.0]), sensor glucose (mean change − 13.2 mg/dL [95% CI − 6.8, 5.0]) and CV (mean change − 0.2% [95% CI − 4.1, 3.8]) (Fig. 1; Tables 2 and 3).

In subjects who reached the 12-month follow-up, we observed a continued improvement in glycemic outcomes with a clinically meaningful increase of TIR from 63% at baseline to 69% (mean change 6.7% [95% CI − 5.5, 18.9]), and reduction of TBR from 0.3% to 0.0% (mean change − 0.3% [95% CI − 0.05, 0.0]), TAR from 36.5% to 30.8% (mean change − 6.4% [95% CI − 18.7, 5.8]), TAR180 from 29.8% to 26.9% (mean change − 3.1% [95% CI − 11.9, 5.8]), and TAR 250 from 6.7% to 3.9% (mean change − 3.2% [95% CI − 8.9, 2.5]) (Fig. 1; Tables 2 and 3).

Moreover, we observed a decrease of GMI from 7.4% at baseline to 7.1% (mean change − 0.2% [95% CI − 0.6, 0.0]), sensor glucose (mean change − 10 mg/dL [95% CI − 26, 6]) and CV (mean change − 1.3% [95% CI − 5.7, 3.2]) (Fig. 1; Tables 2 and 3).

At the 12-month follow-up, seven out of eight subjects achieved all three International Glycemic Targets for this fragile population (TIR > 50%, TBR < 1% and TAR250 < 10%) (18).

### Glycemic outcome during dialysis vs. non-dialysis days

We also compared glycaemic parameters during the dialysis days with the non-dialysis days in all the AID system users, who underwent hemodialysis and had at least one follow-up, considering the last 14 days of sensor data extracted from CareLink™ Clinic.

Out of 11 subjects that underwent hemodialysis and who had at least one follow-up visit, 10 of them (90.9%) underwent dialysis for 6 days out of the 14-days period considered. Only one of them (9.1%) underwent dialysis only for 4 out of the 14 days considered. For the sake of the analysis, glycemic outcomes were stratified first by dialysis vs. non-dialysis day, and then averaged at the patient level.

**Fig. 2:**
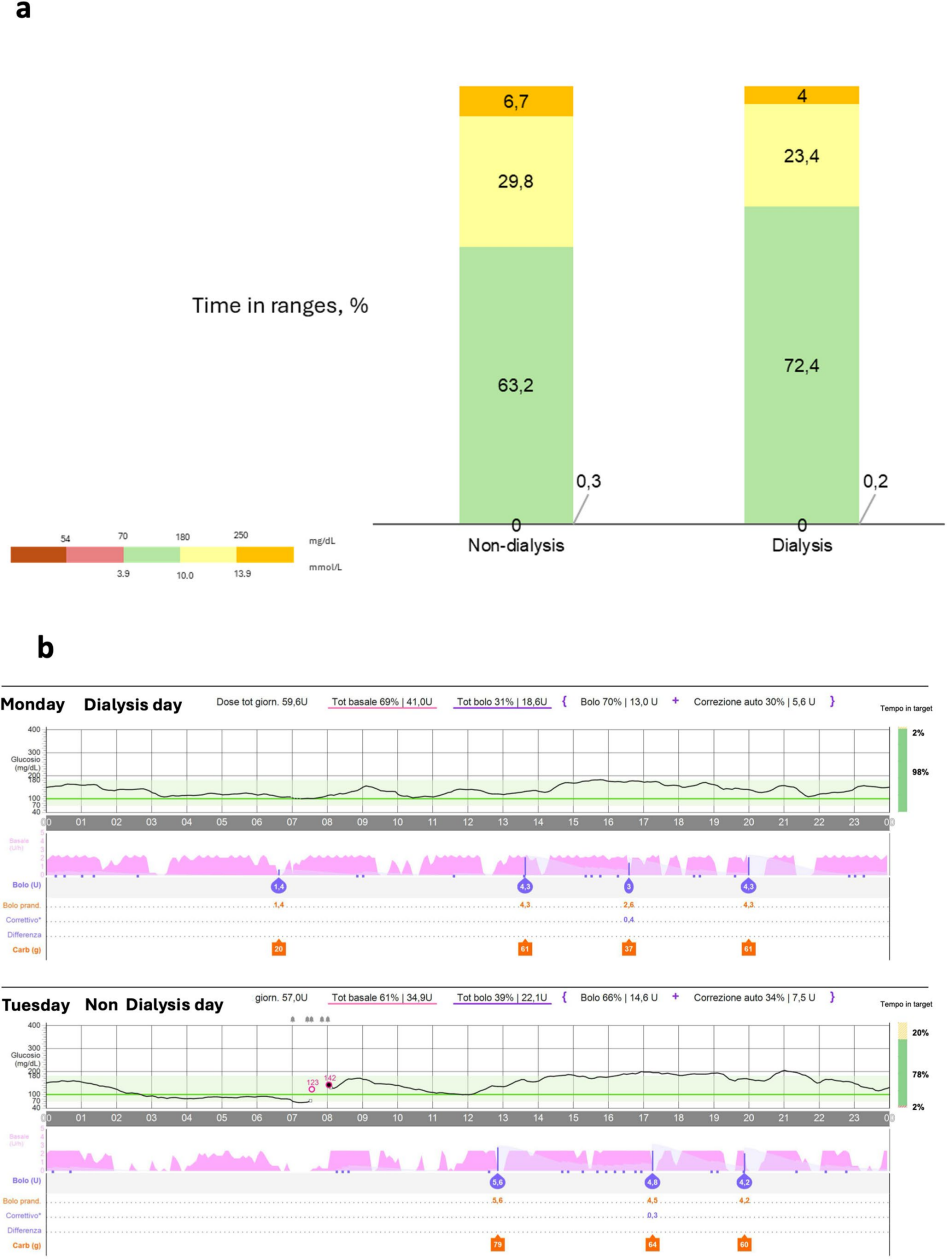
**A** Glycemic outcomes in subjects during non-dialysis and dialysis days. **B** Glycemic report in non-dialysis and dialysis days

We found that TIR was higher (72.4% vs. 63.2%) and TAR was lower (23.4% vs. 29.8%) during the dialysis days compared with non-dialysis days, whereas no differences were observed in TBR (Fig. 2).

### Setting of AID system in subjects undergoing dialysis

All the subjects with diabetes mellitus undergoing dialysis using the AID system considered in this analysis used a glycemic target of 100 mg/dl, an active insulin time (AIT) before and during dialysis of 2.30 h, while no Temporary Target was used during dialysis days.

### Quality of Life evaluation

**Fig. 3:**
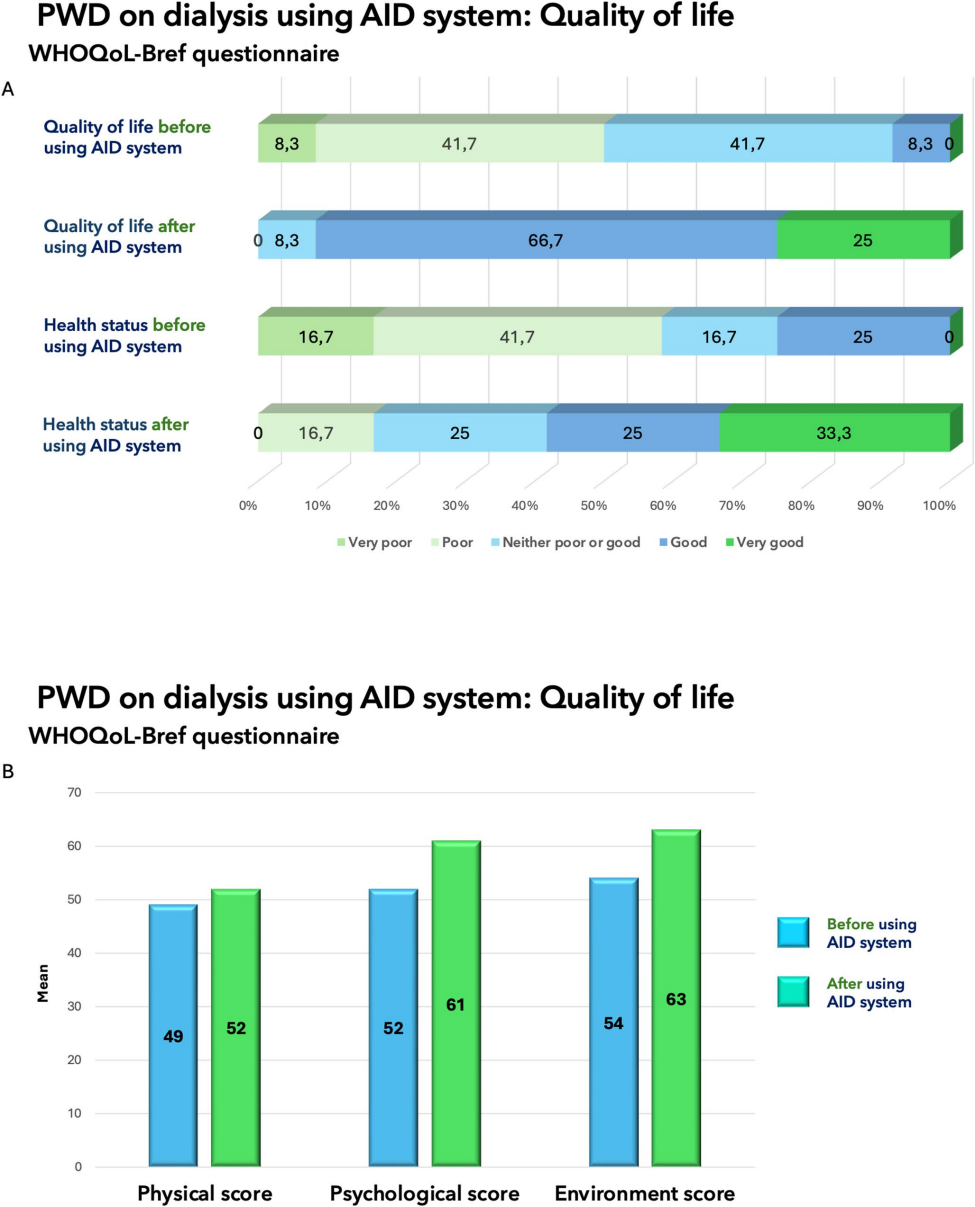
**A**,** B** Quality of life assessed by WHOQoL-Bref questionnaire in dialysis using the AID system

At the last follow-up, all subjects completed the WHOQOL-BREF questionnaire, regardless of the month of follow-up. After starting to use the AID system, 91.7% of the users indicated a good, or very good QOL, whereas only 8.3% had a good QOL and no one had a very good QOL with the previous treatment (Fig. 3a). Moreover, 58.3% of the users were satisfied or very satisfied with their health status versus only 25% before using AID system (Fig. 3a). Additionally, there was an increase in physical, psychological and environment scores in all subjects after use of the AID system compared with the previous treatment (Fig. 3b).

## Discussion

This longitudinal retrospective multicentric pilot real-world study provides evidence that in patients with diabetes undergoing dialysis the use of the AID system is associated with an improvement of glycaemic outcomes and overall QOL. Notably, after just 3 months of use of AID, we observed clinically meaningful improvements in CGM metrics with a decrease of GMI of 0.3%, and an increase of the Time in range of 9.4%. Moreover, we found a clinically relevant reduction of TAR of 9.3% and TBR of 1%. This improvement in glycaemic parameters was maintained over time. Particularly, almost all subjects who reached the 12-month follow-up achieved all three CGM metrics targets recommended for this fragile population [18]. Specifically, we observed a clinically meaninful reduction of TBR from 0.3% to 0.0% over time with no hypoglycaemic episodes detected by CGM or reported by the users.

These findings hold clinical significance as hypoglycaemia presents a substantial obstacle to the optimization of insulin treatment. People with diabetes mellitus undergoing dialysis have high risk of developing severe hypoglycaemic events because of impaired kidney gluconeogenesis, decreased metabolism and clearance of insulin, coexistence of comorbidities (e.g., diabetic gastroparesis and malnutrition), intradialytic glucose shifts into erythrocytes during hemodialysis treatment, use of lower dialysate glucose concentrations over time, and limited access to food during in-center hemodialysis, and frequently exhibit impaired awareness of hypoglycemia [4–7, 19]. Hypoglycemia is even associated with a higher risk of recurrent hypoglycemia and mortality after initiation of dialysis and also imposes a significant burden of care [6].

More generally, glycemic management in patients with dialysis is challenging and individuals have a risk of hyperglycemic crises requiring hospitalization due to increased insulin resistance, impaired insulin secretion, post-dialysis rebound hyperglycemia and a counter-regulatory hormone response in response to the hypoglycemia during the hemodialysis session, and exposure to high dialysate glucose concentrations in those receiving peritoneal dialysis [4, 19]. Hypoglycemic and hyperglycemic episodes that frequently occur during and after dialysis are often unrecognised.

In our study, we had also compared glycaemic parameters during the dialysis days with non-dialysis days. We observed that in all the AID system users who underwent hemodialysis TIR was higher (72.4% vs. 63.2%) and TAR was lower (23.4% vs. 29.8%) during the dialysis days compared with non-dialysis days. Interestingly, no time spent in hypoglycemia was observed during dialysis days. A plausible explanation for the enhanced glycemic control observed could be attributed to the influence of dialysate glucose concentration on glycemic values, leading to an adaptation of the pump’s automatic insulin delivery algorithm. Additionally, dietary intake during dialysis differs from non-dialysis days. Furthermore, the insulin delivery automation of the AHCL system could mitigate post-dialysis hyperglycemia. These findings underscore the significance of an AID system that continuously adapts to glucose fluctuations during and after dialysis treatment, as well as varying insulin requirements from day to day. Moreover, this analysis provides evidence of the safety and the efficacy of the use of ambitious settings (i.e., glycemic target = 100 mg/dL and AIT = 2.30 h), also in vulnerable patients, such as those undergoing dialysis.

Our data confirm and expand the results of two previous studies demonstrating significant improvements in TIR without increasing the risk of hypoglycaemia in adults with T2D in haemodialysis treated with the AID system [16, 17]. Additionally, this study presents evidence in a setting of patients for whom there is limited data on the utilization of AID systems, including, albeit in small numbers, those on dialysis with T1D and one patient undergoing peritoneal dialysis. Furthermore, our findings align with prior evidence demonstrating the attainment of glycemic control targets with the AID system in the general diabetic population [20].

It should be also noted that, at 12 months, in AID users undergoing dialysis, no adverse events and hospitalizations were reported.

It is also truly surprising to note the findings of the enhancement of QOL after the use of the AID system. Notably, the WHOQol-Bref questionnaire submitted to all subjects undergoing dialysis revealed that 91.7% of the AID users indicated a good, or very good QOL, whereas only 8.3% had a good QOL and no one had a very good QOL with the previous treatment. Moreover, 58.3% of the users were satisfied or very satisfied with their health status versus only 25% before using the AID system. Additionally, there was an increase in physical, psychological and environment scores in all subjects after use of the AID system compared with previous treatment. These findings hold greater significance given the substantial patient burden associated with dialysis, particularly considering the presence of numerous comorbidities [6, 7]. The use of the AID system improved QOL independently of improvements in glycaemic control, as it enabled PWD to reduce distress related to hypo and hyperglycaemia, as well as to decrease the number of injections and fingersticks. Indeed, most of the patients had at least one complication; in particular, 71.4% of the subjects had a history of cardiovascular disease, 21.4% had diabetic foot and history of major amputation. It becomes even more important in people with many comorbidities reach glycemic target without hypoglycemia and adverse events and simplify the management of diabetes especially in dialysis days.

Furthermore, the clinical pathway adopted, which consists of a close collaboration between the department of nephrology and diabetologists, was crucial to reduce patients’ burden and improve their acceptance of the new AID therapy; as they were treated only in the dialysis clinic during the whole process of enrollment and follow-up. Our approach could form a model to be followed also in other healthcare systems for treating more patients, improving the management of diabetes mellitus and developing a new standard of care for these people.

This study has some strengths that merit consideration. Our group includes people with T2D and, although fewer in number, also people with T1D on haemodialysis and one patient on peritoneal dialysis, all for whom further investigations on the use of the AID system are needed. Moreover, our analysis includes glycaemic evaluation, also during dialysis sessions. Furthermore, all the analysis and the WHOQol-Bref questionnaires were collected by examiners who were blinded to the clinical data of the study participants.

The present study also has some limitations. First, the retrospective design and the small sample size do not permit any causal inferences. Additionally, the analysis includes only Caucasian individuals, thus limiting the generalizability of the present results to other ethnicities. However, our model of close collaboration between the department of nephrology and diabetologists could be followed also in other healthcare systems. Moreover, the WHOQol-Bref questionnaires, collected only at the last follow-up, may have caused some bias. Further randomized controlled trials and large well-designed real-world studies are needed to confirm these findings.

In conclusion, this analysis has shown how the use of the AID system is safe and can help to improve glycaemic outcomes, with no hypoglycaemia observed in patients with diabetes undergoing hemodialysis. Additionally, the use of the AID system is associated with a consistent improvement of QOL scores in these patients. It is imperative that more attention be paid to the organizational model for the treatment of this fragile population.

## References

[1] IDF Atlas Report (2023) Diabetes and kidney disease. https://diabetesatlas.org/resources/idf-diabetes-atlas-reports/diabetes-a

[2] de Boer IH, Khunti K, Sadusky T, Tuttle KR, Neumiller JN, Rhee CM, Rosas SE, Rossing P, Bakris G (2022) Diabetes management in chronic kidney disease: a consensus report by the American Diabetes Association (ADA) and Kidney Disease: Improving Global Outcomes (KDIGO). Diabetes Care 45:3075–3090. 10.2337/dci22-0027 36189689 10.2337/dci22-0027 PMC9870667

[3] American Diabetes Association Professional Practice Committee (2025) Chronic kidney disease and risk management: standards of care in diabetes-2025. Diabetes Care 48(1 Suppl 1):S239–S251. 10.2337/dc25-S011 39651975 10.2337/dc25-S011 PMC11635029

[4] Galindo RJ, Ali MK, Funni SA, Dodge AB, Kurani SS, Shah ND, Umpierrez GE, McCoy RG (2022) Hypoglycemic and hyperglycemic crises among U.S. adults with diabetes and end- stage kidney disease: population-based study. Diabetes Care 45:2013–2017. 10.2337/dc21-1579 10.2337/dc21-1579 PMC8753755 34740910

[5] Galindo RJ, Beck RW, Scioscia MF, Umpierrez GE, Tuttle KR (2020) Glycemic monitoring and management in advanced chronic kidney disease. Endocr Rev 41:756–774. 10.1210/endrev/bnaa017 32455432 10.1210/endrev/bnaa017 PMC7366347

[6] Chu YW, Lin HM, Wang JJ, Weng SF, Lin CC, Chien CC Epidemiology and outcomes of hypoglycemia in patients with advanced diabetic kidney disease on dialysis: a national cohort study (2017). PLoS ONE 12(3):e0174601. 10.1371/journal.pone.0174601 10.1371/journal.pone.0174601 PMC5371333 28355264

[7] Boughton CK, Hovorka R (2024) The role of automated insulin delivery technology in diabetes. Diabetologia 67:2034–2044. 10.1007/s00125-024-06165-w 38740602 10.1007/s00125-024-06165-w PMC11457686

[8] Mathieu C, Ahmed W, Gillard P, Cohen O, Vigersky R, de Portu S, Ozdemir Saltik AZ (2024) The health economics of automated insulin delivery systems and the potential use of time in range in diabetes modeling: a narrative review. Diabetes Technol Ther 26:66–75. 10.1089/dia.2023.0438 38377319 10.1089/dia.2023.0438

[9] Considine EG, Sherr JL (2024) Real-world evidence of automated insulin delivery system use. Diabetes Technol Ther 26:53–65. 10.1089/dia.2023.0442 38377315 10.1089/dia.2023.0442 PMC10890954

[10] Brown SA, Kovatchev BP, Raghinaru D, Lum JW, Buckingham BA, Kudva YC, Laffel LM, Levy CJ, Pinsker JE, Wadwa RP, Dassau E, Doyle FJ 3rd, Anderson SM, Church MM, Dadlani V, Ekhlaspour L, Forlenza GP, Isganaitis E, Lam DW, Kollman C, Beck RW (2019) iDCL Trial Research Group. Six-month randomized, multicenter trial of closed-loop control in type 1 diabetes N Engl J Med 381:1707–1717. 10.1056/NEJMoa1907863 10.1056/NEJMoa1907863 PMC7076915 31618560

[11] Isganaitis E, Raghinaru D, Ambler-Osborn L, Pinsker JE, Buckingham BA, Wadwa RP, Ekhlaspour L, Kudva YC, Levy J, Forlenza CJ, Beck GP, Kollman RW, Lum C, Brown JW, Laffel SA, iDCL Trial Research Group (2021) Closed-loop insulin therapy improves glycemic control in adolescents and young adults: outcomes from the international diabetes closed-loop trial. Diabetes Technol Ther 23:342–349. 10.1089/dia.2020.0572 33216667 10.1089/dia.2020.0572 PMC8080922

[12] McAuley SA, Lee MH, Paldus B, Vogrin S, de Bock MI, Abraham MB, Bach LA, Burt MG, Cohen ND, Colman PG, Davis EA, Hendrieckx C, Holmes-Walker DJ, Kaye J, Keech AC, Kumareswaran K, MacIsaac RJ, McCallum RW, Sims CM, Speight J, Stranks SN, Sundararajan V, Trawley S, Ward GM, Jenkins AJ, Jones TW, O’Neal DN, Australian (2020) Six months of hybrid closed-loop versus manual insulin delivery with fingerprick blood glucose monitoring in adults with type 1 diabetes: a randomized, controlled trial. Diabetes Care 43:3024–3033. 10.2337/dc20-1447. JDRF Closed-Loop Research Group 33055139 10.2337/dc20-1447

[13] Collyns OJ, Meier RA, Betts ZL, Chan DSH, Frampton C, Frewen CM, Hewapathirana NM, Jones SD, Roy A, Grosman B, Kurtz N, Shin J, Vigersky RA, Wheeler BJ, de Bock MI (2021) Improved glycemic outcomes with Medtronic MiniMed advanced hybrid closed-loop delivery: results from a randomized crossover trial comparing automated insulin delivery with predictive low glucose suspend in people with type 1 diabetes. Diabetes Care 44:969–975. 10.2337/dc20-2250 10.2337/dc20-2250 33579715

[14] Tauschmann M, Thabit H, Bally L, Allen JM, Hartnell S, Wilinska ME, Ruan Y, Sibayan J, Kollman C, Cheng P, Beck RW, Acerini CL, Evans ML, Dunger DB, Elleri D, Campbell F, Bergenstal RM, Criego A, Shah VN, Leelarathna L, Hovorka R (2018) APCam11 Consortium. Closed-loop insulin delivery in suboptimally controlled type 1 diabetes: a multicentre, 12-week randomised trial. Lancet 392:1321–1329. 10.1016/S0140-6736(18)31947-0 30292578 10.1016/S0140-6736(18)31947-0 PMC6182127

[15] Bally L, Gubler P, Thabit H, Hartnell S, Ruan Y, Wilinska ME, Evans ML, Semmo M, Bruno Vogt B, Coll AP, Stettler C, Hovorka R (2019) Fully closed-loop insulin delivery improves glucose control of inpatients with type 2 diabetes receiving hemodialysis. Kidney Int 96:593–596. 10.1016/j.kint.2019.03.006 31133457 10.1016/j.kint.2019.03.006

[16] Boughton CK, Tripyla A, Hartnell S, Daly A, Herzig D, Wilinska ME, Czerlau C, Fry A, Bally L, Hovorka R (2021) Fully automated closed-loop glucose control compared with standard insulin therapy in adults with type 2 diabetes requiring dialysis: an open-label, randomized crossover trial. Nat Med 27:1471–1476. 10.1038/s41591-021-01453-z 34349267 10.1038/s41591-021-01453-z PMC8363503

[17] Phillip M, Nimri R, Bergenstal RM, Barnard-Kelly K, Danne T, Hovorka R, Kovatchev BP, Messer LH, Parkin CG, Ambler-Osborn L, Amiel SA, Lia Bally L, Beck RW, Biester S, Biester T, Blanchette JE, Bosi E, Boughton CK, Breton MD, Brown SA, Buckingham BA, Cai A, Carlson AL, Castle JR, Choudhary P, Close KL, Cobelli C, Criego AB, Davis E, de Beaufort C, de Bock MI, DeSalvo DJ, DeVries JH, Dovc K, Doyle FJ, Ekhlaspour L, Shvalb NF, Forlenza GP, Gallen G, Garg SK, Gershenoff DC, Gonder-Frederick LA, Haidar A, Hartnell S, Lutz Heinemann L, Heller S, Hirsch IB, Hood KK, Isaacs D, Klonoff DC, Kordonouri O, Kowalski A, Laffel L, Lawton J, Lal RA, Leelarathna L, Maahs DM, Murphy HR, Nørgaard K, O’Neal D, Oser S, Oser T, Renard E, Riddell MC, Rodbard D, Russell SJ, Schatz DA, Shah VN, Sherr JL, Simonson GD, Wadwa RP, Ward C, Weinzimer SA, Wilmot EG, Battelino T Consensus Recommendations for the Use of Automated Insulin Delivery Technologies in Clinical Practice (2023). Endocr Rev 4;44:254–2580. 10.1210/endrev/bnac022 10.1210/endrev/bnac022 PMC9985411 36066457

[18] Battelino T, Danne T, Bergenstal RM, Amiel SA, Beck R, Bioester T, Bosi E, Buckingham BA, Cefalu WT, Close KL, Cobelli C, Dassau E, DeVries JH, Donaghue KC, Dovc K, Doyle FJ 3rd, Garg S, Grunberger G, Heller S, Heinemann L, Hirsch IB, Hovorka R, Jia W, Kordonouri O, Kovatchev B, Kowalski A, Laffel L, Levine B, Mayorov A, Mathieu C, Murphy HR, Nimri R, Nørgaard K, Parkin CG, Renard E, Rodbard D, Saboo B, Schatz D, Stoner K, Urakami T, Weinzimer SA, Phillip M (2019) Clinical targets for continuous glucose monitoring data interpretation: recommendations from the international consensus on time in range. Diabetes Care 42:1593–1603. 10.2337/dci19-0028 31177185 10.2337/dci19-0028 PMC6973648

[19] Galindo RJ, Soliman D, Cherñavvsky D, Rhee CM (2024) Diabetes technology in people with diabetes and advanced chronic kidney disease. Diabetologia 67:2129–2142. 10.1007/s00125-024-06244-y 39112642 10.1007/s00125-024-06244-y PMC11446991

[20] Choudhary P, Arrieta A, van den Heuvel T, Castañeda J, Smaniotto V, Cohen O (2024) CCelebrating the data from 100,000 Real-World users of the MiniMed™ 780G system in Europe, Middle East, and Africa collected over 3 Years: from data to Clinical Evidence. Diabetes Technol Ther 26:32–37. 10.1089/dia.2023.0433 38377326 10.1089/dia.2023.0433 PMC10890936

